# Depletion of m^6^A reader protein YTHDC1 induces dilated cardiomyopathy by abnormal splicing of *Titin*

**DOI:** 10.1111/jcmm.16955

**Published:** 2021-10-30

**Authors:** Siyun Gao, Haifeng Sun, Kejing Chen, Xueying Gu, Hongyu Chen, Liudan Jiang, Lei Chen, Shengqi Zhang, Yi Liu, Dan Shi, Dandan Liang, Liang Xu, Jian Yang, Yanjiao Ruan, Hao Chen, Bin Shen, Honghui Ma, Yi‐Han Chen

**Affiliations:** ^1^ Department of Cardiology Shanghai East Hospital Tongji University School of Medicine Shanghai China; ^2^ Key Laboratory of Arrhythmias of the Ministry of Education of China Shanghai East Hospital Tongji University School of Medicine Shanghai China; ^3^ State Key Laboratory of Reproductive Medicine Center for Global Health Gusu School Women’s Hospital of Nanjing Medical University Nanjing Maternity and Child Health Hospital Nanjing Medical University Nanjing China; ^4^ Institute of Medical Genetics Tongji University Shanghai China; ^5^ Department of Pathology and Pathophysiology Tongji University School of Medicine Shanghai China; ^6^ School of Medicine Southern University of Science and Technology Shenzhen China; ^7^ Research Units of Origin and Regulation of Heart Rhythm Chinese Academy of Medical Sciences Shanghai China

**Keywords:** dilated cardiomyopathy, epitranscriptomics, heart failure, RNA modification, YTHDC1

## Abstract

*N* ^6^‐methyladenosine (m^6^A) is the most prevalent modification in mRNA and engages in multiple biological processes. Previous studies indicated that m^6^A methyltransferase METTL3 (‘writer’) and demethylase FTO (‘eraser’) play critical roles in heart‐related disease. However, in the heart, the function of m^6^A ‘reader’, such as YTH (YT521‐B homology) domain‐containing proteins remains unclear. Here, we report that the defect in YTHDC1 but not other YTH family members contributes to dilated cardiomyopathy (DCM) in mice. Cardiac‐specific conditional *Ythdc1* knockout led to obvious left ventricular chamber enlargement and severe systolic dysfunction. YTHDC1 deficiency also resulted in the decrease of cardiomyocyte contractility and disordered sarcomere arrangement. By means of integrating multiple high‐throughput sequence technologies, including m^6^A‐MeRIP, RIP‐seq and mRNA‐seq, we identified 42 transcripts as potential downstream targets of YTHDC1. Amongst them, we found that *Titin* mRNA was decorated with m^6^A modification and depletion of YTHDC1 resulted in aberrant splicing of *Titin*. Our study suggests that *Ythdc1* plays crucial role in regulating the normal contractile function and the development of DCM. These findings clarify the essential role of m^6^A reader in cardiac biofunction and provide a novel potential target for the treatment of DCM.

## 1 INTRODUCTION

*N* ^6^‐methyladenosine (m^6^A) is the most abundant internal epigenetic modification on eukaryotic mRNAs.^1^, ^2^, ^3^ Over 12,000 m^6^A sites characterised by a typical consensus in the transcripts of more than 7000 human genes have been identified.^4^ Built on the understanding of its widespread prevalence on mRNA, m^6^A has been shown to play crucial roles in mRNA fate determination and participates in multiple biological processes. Recent studies uncovered that m^6^A modification serves to facilitate critical steps in mRNA splicing, translation initiation and decay.^5^, ^6^

Similar to epigenetic marks on DNA and histone, m^6^A on RNA is also dynamic and reversible. The m^6^A modification is installed by the METTL3‐METTL14‐WTAP methyltransferase ‘writer’ complex^7^ and can be removed by m^6^A demethylases FTO^8^ and ALKBH5 (‘eraser’).^9^ Both the ‘writer’ and ‘eraser’ are essential in pathological remodelling of the heart. METTL3 deficiency in mice induces maladaptive remodelling and ultimately causes heart failure,^10^ whilst reduced FTO expression is observed in failing mammalian hearts, which may attenuate the ischaemia‐induced cardiac remodelling.^11^ These results documented the importance of m^6^A modification in the aetiology of cardiac disease.

Whilst methyltransferases and demethylases act as the ‘writer’ and ‘eraser’ of m^6^A on mRNA, respectively, the downstream effects of RNA m^6^A modification are specifically deciphered by the ‘reader’ YTH (YT521‐B homology) domain‐containing proteins, including cytoplasmic YTHDF1–YTHDF3,^12^, ^13^, ^14^ YTHDC2^15^ and nuclear YTHDC1.^16^, ^17^ In the cytosol, YTHDF1 and YTHDF3 work in concert to enhance the translation of target mRNAs,^12^, ^14^ whilst YTHDF2 has been shown to affect the stability of m^6^A‐modified RNAs by localising them to mRNA decay machinery.^13^ YTHDC2 either promotes the translation efficiency of its targets or decreases RNA stability by interacting with different binding partners.^18^ Intriguingly, as the sole nuclear m^6^A reader, YTHDC1 modulates nuclear processing of its targets including alternative splicing (AS),^16^ export of m^6^A decorated mRNA^19^ and turnover of chromatin‐associated RNA.^20^, ^21^ Functional deficit of YTH family proteins can induce multiple diseases. However, the exact roles of m^6^A readers in heart are poorly understood.

Dilated cardiomyopathy (DCM) is one of the most common causes of heart failure and indication for heart transplantation worldwide.^22^ An estimated 35%–40% of genetic DCMs may result from sarcomere gene mutations.^23^, ^24^ Of the known genetic mutations that cause DCM, *Titin* (TTN) mutations account for 20%–25% of cases.^25^ There are two major *Titin* mRNA isoforms, N2BA and N2B. The increased ratio of N2BA:N2B due to the abnormal *Titin* pre‐mRNA splicing can directly lead to DCM.^26^ However, the regulatory molecular mechanisms of *Titin* splicing are still largely unknown.

In this work, we reported that amongst the four major YTH domain‐containing proteins, only cardiac‐specific ablation of *Ythdc1* contributes to a typical DCM phenotype in mice. The loss of YTHDC1 leads to aberrant splicing of Titin, inducing an increased ratio of N2BA:N2B isoform, which finally leads to DCM.

## 2 MATERIAL AND METHODS

The data that support the findings of this study are available from the corresponding authors upon reasonable request.

### 2.1 Mouse models and anaesthesia

This study conformed to the rules of the Guide for the Care and Use of Laboratory Animals made by the U.S. National Institutes of Health. All the animal experiments were approved by the Animal Care and Use Committee of Tongji University School of Medicine.

**FIGURE 1:**
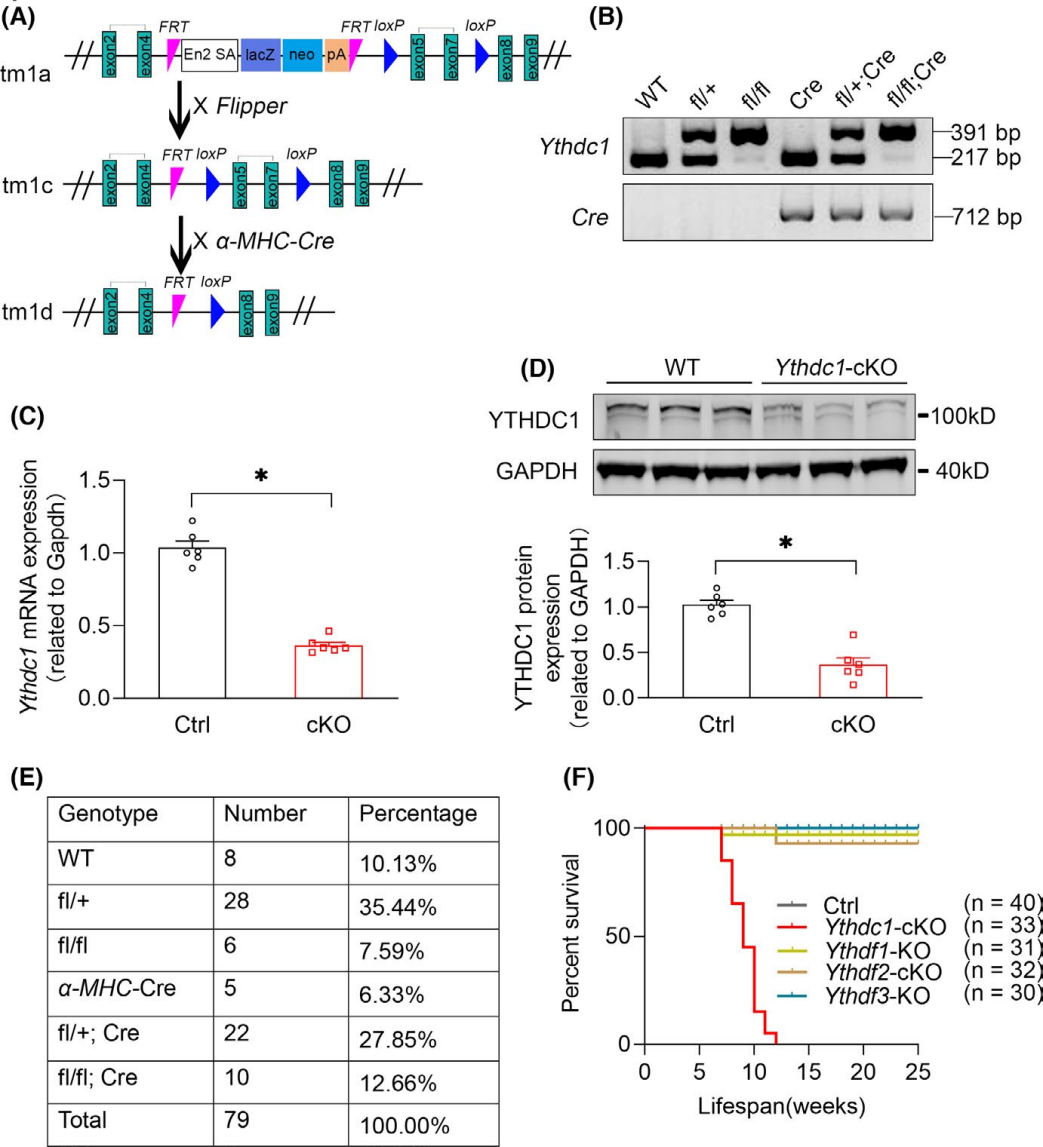
Generation of cardiac‐specific YTHDC1 knockout mice. (A) Schematic diagram represents WT and mutant loci of *Ythdc1* gene together with the targeting vector. Exons for the gene encoding *Ythdc1* were represented by a green box. Conditional *Ythdc1* knockout mice were generated by cross‐breeding *Ythdc1* loxP/WT mice with *α*‐*mhc*‐*cre* positive mice. (B) PCR genotyping analysis of *Ythdc1* knockout mice. *Ythdc1* ^−/−^ produced the expected 391 bp mutant fragment compared with a 217 bp wild‐type fragment. (C) qPCR analysis of *Ythdc1* expression in WT and *Ythdc1*‐cKO mice left ventricle. The data are shown as means ±SEM *n* = 6. * *p* < 0.01 compared with ctrl by unpaired *t* test. (D) Typical Western blot analysis of YTHDC1 expression in WT and *Ythdc1*‐cKO mice left ventricle. The pooled data below are shown as means ±SEM *n* = 6. *p = 0.014 compared with ctrl by unpaired *t* test. (E) Viable mice were born at approximately Mendelian ratios. (F) Cumulative survival curve of Ctrl (*n* = 40), *Ythdc1*‐cKO (*n* = 33), *Ythdf1*‐KO (*n* = 31), *Ythdf2*‐cKO (*n* = 32) and *Ythdf3*‐KO (*n* = 30) mice. Data are presented as mean ± SEM

All the generation construction strategies of the YTH domain‐containing proteins were shown in Figure 1A and Figure S1A–S1C. Importantly, the conditional *Ythdc1* targeted mice were generated by inserting the loxP sites covering the exon 5 to exon 7. To disrupt the *Ythdc1* gene in cardiomyocytes, *Ythdc1* knockout (KO) mouse embryonic stem cell clone was purchased from CAM‐SU GRC, microinjected into mouse blastocysts and implanted into pseudo‐pregnant mice. The resulting chimeric mice were crossed with FLPeR mice to excise the FRT flanked selection cassette to obtain *Ythdc1* flox mice which were then crossed with *α*‐*MHC*‐*Cre* transgenic mice to generate *Ythdc1* conditional knockout (cKO) mice. All mice used in this study were maintained in C57BL/6J genetic background. Primer sequences for genotyping are listed in Table S1.

### 2.2 Quantitative real‐time PCR (qRT‐PCR)

Total RNA was extracted from mouse left ventricles using TRIzol reagent (T9424, Sigma) according to the manufacturer's recommendations. 0.5 μg of total RNA was reverse‐transcribed into cDNA with PrimeScript Reverse Transcriptase (2680B, Takara). qRT‐PCR was performed by using SYBR Green Supermix (636600, Toyobo) in a Thermo QuantStudio 6 Flex Real‐Time PCR system. Gene expression was determined relative to *Gapdh* using the log_2_ fold change method. All qRT‐PCR primers covered exon‐exon junctions when possible. Primer sequences for qRT‐PCR are listed in Table S1.

### 2.3 Western blot

Western blot was performed as described previously.^27^ Briefly, tissue homogenates were lysed in RIPA buffer (50 mM Tris, 10 mM EDTA, 150 mM NaCl, 0.25% deoxycholic acid, 0.1% SDS, 1% NP‐40 substitute) with protease inhibitors (Roche). After centrifugation, the supernatant was collected and protein was separated on 10% SDS‐polyacrylamide gel electrophoresis and transferred to PVDF membrane. The membranes were blocked with 3% bovine serum albumin and incubated with the following primary antibodies: YTHDC1 (ab220159,Abcam), GAPDH (60004,Proteintech) overnight at 4°C. The secondary antibodies conjugated to infrared dyes (LI‐COR Biosciences) were applied, and the blots were visualised with an Odyssey imager and quantified by ImageJ software.

### 2.4 TITIN protein isoform analysis

Protein samples from left ventricles were homogenised in sample buffer (8 M urea, 2 M thiurea, 0.05 M Tris pH 6.8, 75 mM DTT, 3% SDS, 0.05% bromophenol blue), separated on an SDS/agarose gel electrophoresis system^28^and Coomassie‐stained to visualise the TITIN isoforms N2BA (several sizes, including both the N2A and N2B regions), N2B and the proteolytic fragment T2.

### 2.5 Histology

Hearts were dissected from age‐ and sex‐matched littermates, washed in PBS and fixed overnight in 4% paraformaldehyde. Samples were subsequently dehydrated in 70% ethanol, embedded in paraffin and coronally sectioned (8‐μm thick). Sections were stained with haematoxylin and eosin according to previously published methods.^27^

### 2.6 Transmission electron microscopy (TEM)

Fresh left ventricular tissues were carefully kept in a relaxed and slightly stretched state during sample preparation and sectioned into 1 mm tissue blocks, then immediately immersed in ice‐cold 2.5% glutaraldehyde to fix 1 h, at 4°C. Blocks were repeatedly washed in phosphate buffer and postfixed in 1% osmium tetroxide for 1 h. Samples were dehydrated in increasing concentrations of ethanol, starting with 10%, to 50%, 70% and 90% of ethanol, then embedded in 100% epoxy resin and left to polymerize at 55°C in 5% CO2 for 36 h. The resin blocks were then sectioned with an ultramicrotome. The ultrathin sections were placed on the grids, stained with uranyl acetate and lead citrate solution for TEM observation (JOEL TEM1230).

### 2.7 Adult cardiomyocyte isolation

Cardiomyocytes were isolated as previously described,^27^ with minor modification. 8‐week‐old male mice were injected with 200 μl of heparin (100 IU/ mouse) before being anaesthetized with pentobarbital (70 mg/kg). Hearts were dissected and placed in a Langendorff apparatus, perfused with Ca^2+^‐free Tyrode buffer pH 7.4 (in mM: NaCl, 113; KCl, 4.7; KH2PO4, 0.6; Na2HPO4, 0.6; MgSO4, 1.2; HEPES, 10; NaHCO3, 12; KHCO3, 10; taurine, 30; butanedione monoxime, 10; glucose, 5.5) for 3 min, then digested with collagenase type II (300 U/ml, Worthington) for 20 min. When the hearts became slightly pale and flaccid, the ventricles were removed, cut into small pieces in modified Kraftbruhe (KB) solution (100 mM potassium glutamate, 10 mM potassium aspartate, 25 mM KCl, 10 mM KH_2_PO_4_, 2 mM MgSO_4_, 20 mM taurine, 5 mM creatine, 0.5 mM EGTA, 20 mM glucose, 5 mM HEPES and 1.0% BSA (pH was adjusted to 7.2 with KOH), filtered through a cell strainer (100 µm, BD Falcon) and centrifuged at 4000 × *g* for 3 min. After removing supernatant, the cell pellet was resuspended in 5 ml KB solution. For electrophysiological experiments, gradient re‐calcification for the acute isolated cells was performed by using 1 mM Ca^2+^ solution per 30 min to a final concentration of 1.8 mM.

### 2.8 Cardiomyocyte contractility measurements

Cardiomyocyte contractility was measured as previously described.^29^ Isolated cardiomyocytes were placed into a thermostatically controlled chamber with oxygenated Krebs balanced salt solution (pH 7.4) containing dextrose and Ca^2+^ (1 mmol/L) at 37°C and imaged with an inverted microscope. Individual cardiomyocytes were selected for analysis on the basis of a characteristic rod‐shaped morphology with no membrane blebbing and quiescence in the presence of extracellular Ca2^+^ (l mmol/L). The changes in light intensity at the cell edges were used to track cell motion. All parameters were calculated for each contraction, and the results were shown as average of all observed contractions.

### 2.9 Echocardiography

Cardiac function was assessed by serial echocardiography.^30^ Left ventricular (LV) systolic function was evaluated at the indicated time points by echocardiography (Visual Sonics Vevo 770) under conditions of 1% isoflurane anaesthesia with spontaneous ventilation. Two‐dimensional B‐mode imaging was used to capture the long‐axis projection with guided M‐mode images. The left ventricular ejection fraction (LVEF) and left ventricular internal diameter (LVID) were calculated based on end‐diastolic and end‐systolic dimensions obtained from M‐mode ultrasound.

### 2.10 Isolation and transfection of neonatal rat ventricular myocytes.

Ventricles from neonatal rats were separated from the atria, cut into small pieces and then dissociated in Ca^2+^‐free HBSS containing 0.125 mg/ml trypsin (Gibco), 10 mg, ml DNase II (Sigma) and 0.1 mg/ml collagenase type IV (Sigma). Digestion was performed at 37°C by stirring the digestion solution containing the heart sections throughout the repeated 5‐min period of digestion for 8–10 times. The supernatant was collected with FBS (Gibco) after each digestion period to terminate the digestion. The cell pellets were resuspended in DMEM (Gibco) supplemented with 10% FBS and with 100 mM 5‐bromo‐20‐deoxyuridine (Sigma) and seeded onto 100‐mm plastic dishes for 2 h at 37°C in a 5% CO_2_ and humidified atmosphere. The supernatant was the plated onto 1% gelatin (Sigma)‐coated dishes. Twenty‐four hours after the seeding, the medium was changed to DMEM (Gibco) containing 2% FBS (Gibco), 1% insulin‐transferrin‐selenium (ITS; Gibco), 1% penicillin‐streptomycin (Gibco) and 100 mM5‐bromo‐20‐deoxyuridine (Sigma). For the gene silencing studies, the siRNA against negative control (NC) and *Ythdc1* (50 nM) were transfected using Lipofectamine RNAiMAX (Invitrogen) after 72 h of cardiomyocytes seeding and were used to conduct the further experiments.

### 2.11 Microelectrode array (MEA) recording and analysis

The cardiomyocytes were maintained in cardiac medium without FBS for at least 24 h, and then the cells were dissociated and resuspended in cardiac recovery medium at a density of 4 x 10^6^ cells/mL. Aliquots of the cell suspensions (10 μl) were plated on CytoView MEA 24 plates (Axion BioSystems). The MEA device automatically adjusted and controlled the environment (37°C and 5% CO_2_) to maintain the temperature and pH of the medium. Data were acquired using the Maestro Pro multi‐well MEA platform (Axion BioSystems) and analysed by the Maestro cardiac analysis tool (Axion BioSystems).

### 2.12 Ribo‐minus RNA sequencing (RNA‐seq) library preparation

Total RNAs from left ventricular tissues of 2‐week‐old and 8‐week‐old male mice were extracted to construct RNA‐seq libraries. RNA samples were pre‐treated with RiboMinus Eukaryote Kit (A15020, Thermo Fisher) to remove ribosomal RNAs. The remaining intact RNA was fragmented, and RNA‐seq libraries were prepared by using a NEB Ultra II Directional RNA Library Prep Kit (E7760, NEB). End‐repaired fragments were ligated with a unique Illumina adapter.

### 2.13 m^6^A sequencing (m^6^A‐seq)

m^6^A‐seq and library preparation were conducted according to a previously published protocol with following modifications.^4^ Briefly, 100 μg total RNAs were extracted from left ventricular tissues of 8‐week‐old male mice using TRIzol following the manufacturer's protocol. Fragmented mRNAs were immunoprecipitated with anti‐m^6^A antibody (56593, CST) in IP buffer (150 mM NaCl, 0.1% NP‐40, and 10 mM Tris‐HCl, pH 7.4) for 3 h, at 4 °C, and 1/10 of the fragmented mRNA was saved as input. The antibody‐RNA complex was isolated by incubation with protein G beads (10004D, Thermo Fisher) for 2 h, at 4 °C. The beads were washed three times and eluted competitively with m^6^A monophosphate solution. The RNA‐seq libraries were prepared using a NEB Ultra II Directional RNA Library Prep Kit (E7760, NEB).

### 2.14 YTHDC1 RNA immunoprecipitation sequencing (RIP‐seq)

Total RNAs from left ventricular tissues of 2‐week‐old male mice were extracted to construct YTHDC1 RIP‐seq libraries. A Magna RIP RNA‐Binding Protein Immunoprecipitation Kit (Millipore) was used to acquire specific RNA targets according to the manufacturer's instruction. YTHDC1 antibody (ab220159, Abcam) was used for the RIP assays. The YTHDC1‐binding RNA was purified from the protein‐RNA complex by acid‐phenol‐chloroform extraction. Ribosomal RNAs were removed with RiboMinus Eukaryote Kit (A15020, Thermo Fisher). The cDNA library was constructed with the NEBNext Ultra II Directional RNA Library Prep Kit for Illumina (E7760, NEB).

### 2.15 Sequencing data analysis

General pre‐processing of reads: all samples were sequenced by Illumina HiseqX10 or Hiseq4000 with paired‐end 150bp reads generated for analysis. Reads were quality controlled using fastqc and aligned to GRCm38 using HISAT2^31^ with default parameters. All bigWig files were obtained by using bamCoverage in deepTools.^32^

For RNA‐Seq, read counts were quantified using feature counts.^33^ Differential expression testing was performed with R package DESeq2.^34^ Genes with adjusted *P* values less than 0.05 and Fold Change >1.5 were marked as differentially expressed genes (DE genes). AS analysis was performed by using rMATS.^35^ AS events with FDR <0.05 and | IncLevelDifference | >0.05 were marked as significant. Gene ontology (GO) enrichment analysis was performed on Metascape.^36^ For RIP‐Seq, genes with adjusted *p* values less than 0.05 and Fold Change >1.2 were identified as YTHDC1 targets. For m^6^A‐Seq, m^6^A peaks were called by using exomePeak^37^ with default parameters. Peaks annotation was performed with ChIPseeker,^38^ and m^6^A motif was called by findMotifsGenome in Homer.^39^ Metagene plot of m^6^A was performed by using Guitar.^40^ Venn plots were conducted with TBTools^41^ or R package eulerr. Other plots were obtained by using R package eulerr. All data generated or analysed during this study are included either in this article or in the supplemental information files.

### 2.16 Statistics

All the data were presented as mean ±SEM. All statistical analyses were carried out using GraphPad Prism 8. Two‐tailed Student's *t* test or nonparametric Mann‐Whitney test was used for comparisons between two groups, and two‐way ANOVA was used to compare the difference amongst multiple groups. Statistical significance was defined as a *p* value less than 0.05.

## 3 RESULTS

### 3.1 Generation of cardiac‐specific *Ythdc1* conditional knockout mice

To understand the functional involvement of *Ythdc1* in postnatal heart and potential contribution to heart diseases, we generated a conditional deletion allele of the mouse *Ythdc1* gene. Two loxP sites were engineered to flank a region of *Ythdc1* genomic DNA encoding exons 5–7. Mice carrying conditional *Ythdc1* allele were crossed with mice carrying the Cre recombinase gene driven by the α‐Mhc promoter (Figure 1A), which expresses Cre recombinase in heart after birth. Cre‐mediated recombination deleted *Ythdc1* exon 5–7 between the loxP sites. This recombination was confirmed in the DNA of the *Ythdc1*‐cKO mice. As shown in Figure 1B, homozygous *Ythdc1*‐cKO mice were given the expected 391 bp mutant fragment compared with a 217 bp wild‐type (WT) fragment. The RT‐qPCR and Western blot analysis were used to evaluate the gene knockout efficiency of Ythdc1 at mRNA and protein levels, respectively.

The results showed a significant reduction of *Ythdc1* expression in cKO mice both in mRNA level (Figure 1C) and in protein level (Figure 1D). Despite the newborn homozygous *Ythdc1*‐cKO mice were viable and exhibited the expected mendelian ratios (Figure 1E), all of them dead in around 10 weeks (Figure 1F).

Likewise, to know the rest m^6^A ‘reader’ proteins’ function in heart, we also generated the other three genetic knockout mice, including *Ythdf1*‐KO, *Ythdf2*‐cKO and *Ythdf3*‐KO (Figure S1A–S1C), to allow assessment the role of each m^6^A ‘reader’ protein family member in cardiac function. We did not include *Ythdc2*, as its expression is barely detected in heart. As shown in Figure S2, the reduction level of *Ythdf1* (Figure S2A, S2B), *Ythdf2* (Figure S2C¸ S2D) and *Ythdf3* (Figure S2E, S2F) was also assured by Western blot. Amongst all the four mice models, we found that only *Ythdc1*‐*cKO* mice were extremely susceptible to premature death, whilst littermates of the rest YTH family members did not display abnormal phenotype after knockout (Figure 1F). These results implicated that YTHDC1 plays an essential role in postnatal heart.

### 3.2 Loss of cardiac *Ythdc1* in the heart leads to DCM

**FIGURE 2:**
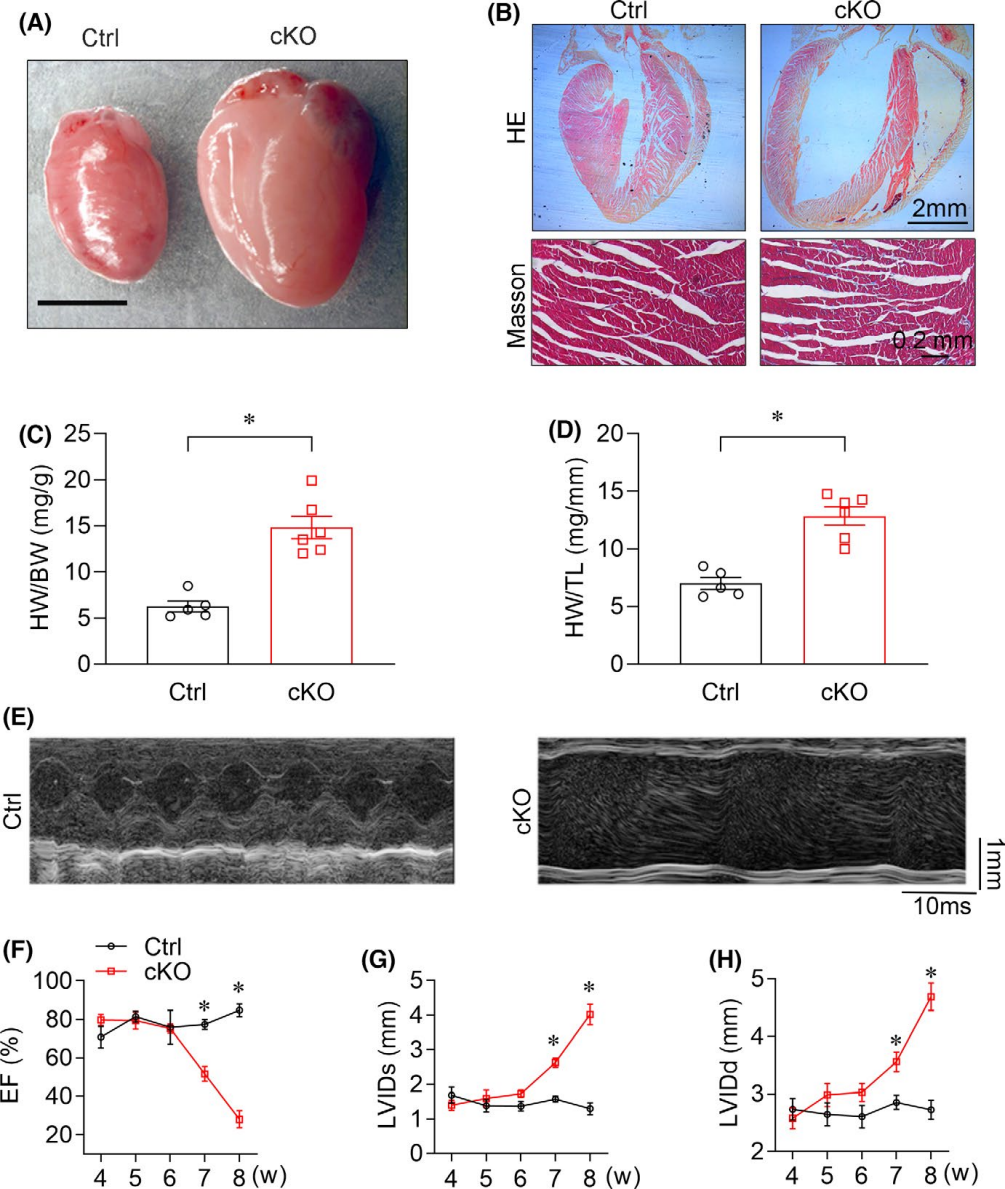
Cardiac‐specific YTHDC1 deletion leads to DCM. (A) Representative microscopic views of Ctrl and *Ythdc1*‐cKO entire mouse hearts (scale bar: 5 mm). (B) Top, Representative H&E staining of Ctrl and *Ythdc1*‐cKO heart paraffin‐sections (scale bar: 2 mm). Bottom, Representative Masson staining of Ctrl and *Ythdc1*‐cKO heart (scale bar: 0.2 mm). (C)–(D) Ratio of HW to BW (*p < 0.001) and HW to TL (* *p* < 0.001) for control (*n* = 5) versus cKO (*n* = 6) mice at 8 weeks of age. The adjusted *p*‐value was calculated by the unpaired *t* test. (E) Representative echocardiographic images of Ctrl and cKO mice at 8‐week‐old (scale bar: 1 mm). (F)–(H) Echocardiographic measurements for control and cKO mice of LVEF, LVIDd and LVIDs. (*n* = 5–6 mice at 4, 5, 6, 7 and 8 weeks of age, the adjusted *p*‐value was calculated by the repeated‐measures two‐way ANOVA test). Data are represented as the mean ±SEM *p < 0.05. LVEF, left ventricular ejection fraction; LVIDd left ventricular internal diameter at end‐diastole; LVIDs, left ventricular internal diameter at end‐systole

To interrogate the exact cause of premature death, we evaluated the hearts by cardiac histopathology and echocardiography on *Ythdc1*‐cKO mice. Gross examination and histological analysis of cKO hearts revealed apparent cardiac enlargement in live 8‐week‐old cKO mice (Figure 2A, 2B). The Masson staining showed that cardiac fibrosis was increased in 8‐week‐old *Ythdc1*‐cKO mice (Figure 2B). Consistently, both the profibrotic genes (*Col1a1* and *Col3a1*) were significantly increased in the hearts in 8‐week‐old cKO mice (Figure S3A). Although there was no body weight (BW) loss in cKO mice, the ratios of heart weight (HW) to BW (HW/BW) (Figure 2C) and HW to tibia length (TL) (HW/TL) (Figure 2D) were significantly increased in cKO mice at 8 weeks of age, implicating decompensated heart failure.

We then monitored cardiac function with echocardiography from week 4 to 8 after birth. Consistent with pathological observations, as early as week 6, an age‐dependent reduction in LV systolic function (Figure 2E, 2F) and increase in both end‐diastolic and end‐systolic LV internal diameter (LVIDd and LVIDs) began to emerge in YTHDC1 deficiency mice (Figure 2G, 2H). These data suggested that *Ythdc1*‐*cKO* mice developed typical manifestation of DCM, such as obvious left ventricular chamber enlargement and severe systolic dysfunction.

### 3.3 **YTHDC1 deficiency attenuates the contraction of cardiomyocytes**.

**FIGURE 3:**
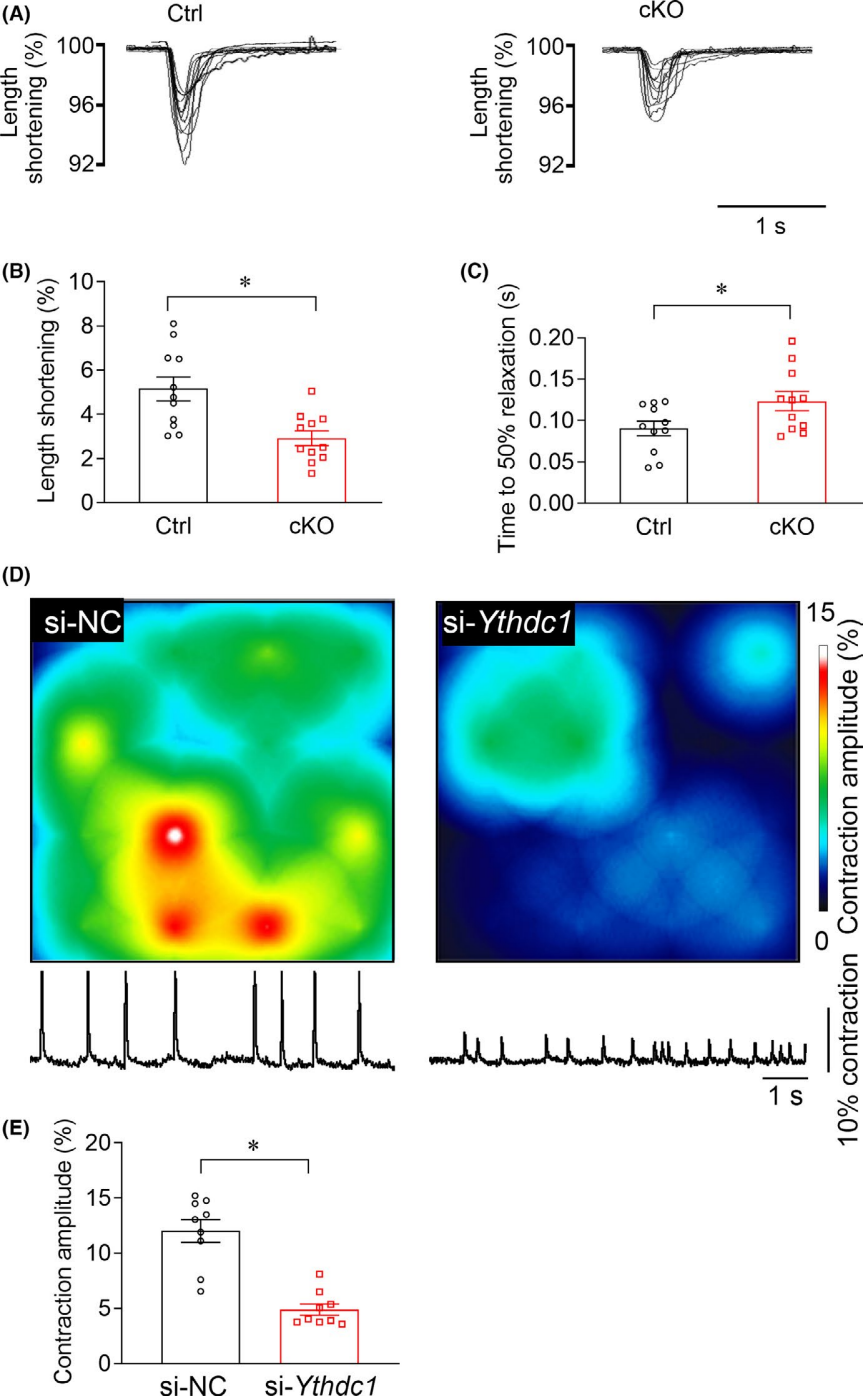
YTHDC1 deficiency attenuates the contractility of cardiomyocytes. (A) Cardiomyocytes contraction depicting cell shortening in cardiomyocytes isolated from Ctrl and *Ythdc1*‐cKO group. (B)–(C) Time to 50% relaxation durations (*p* = 0.034) and cell length shortening (*p* < 0.001) in Ctrl and *Ythdc1*‐cKO mouse cardiomyocytes taken from three hearts per genotype. (D) Multichannel electrophysiological electrode array for extracellular recording of the cardiac myocytes conduction for the si‐NC and si‐*Ythdc1* group. Up, conduction amplitude heat map of the cardiomyocyte monolayer with 16 electrodes. Down, the typical trace of the cardiomyocyte contraction on the electrodes. (E) Pooled data of the contraction amplitude from D. The data are shown as means ±SEM. *n* = 9. * *p* < 0.001 compared with si‐NC by unpaired *t* test

Considering that inability to produce sufficient contraction is the hallmark of DCM and heart failure, we measured the contractility of single cardiomyocytes isolated from the ventricle of 8‐week‐old Ctrl and cKO mice (Figure 3A). We observed that YTHDC1 deficiency significantly prolonged the relaxing time of cardiomyocytes (Figure 3B) and reduced the amplitude of length shortening (Figure 3C).

To further confirm the finding in *Ythdc1* deficiency mice and circumvent the off‐target effects of the CRISPR gene knockout strategy, we subsequently measured the conduction of cultured cardiomyocytes through the multiple microelectrode array (MEA) recording. We first determined the efficiency of gene knockdown by Western blots, confirming that more than 50% reduction of YTHDC1 expression at protein level (Figure S4A, S4B). And then we found that knockdown of YTHDC1 significantly decreased the contraction amplitude of the cardiomyocyte monolayers (Figure 3D, 3E), implying that YTHDC1 is critical for regulating the contraction of cardiomyocytes.

### 3.4 Multidimensional sequencing identifies *Titin* as a direct downstream target of YTHDC1

**FIGURE 4:**
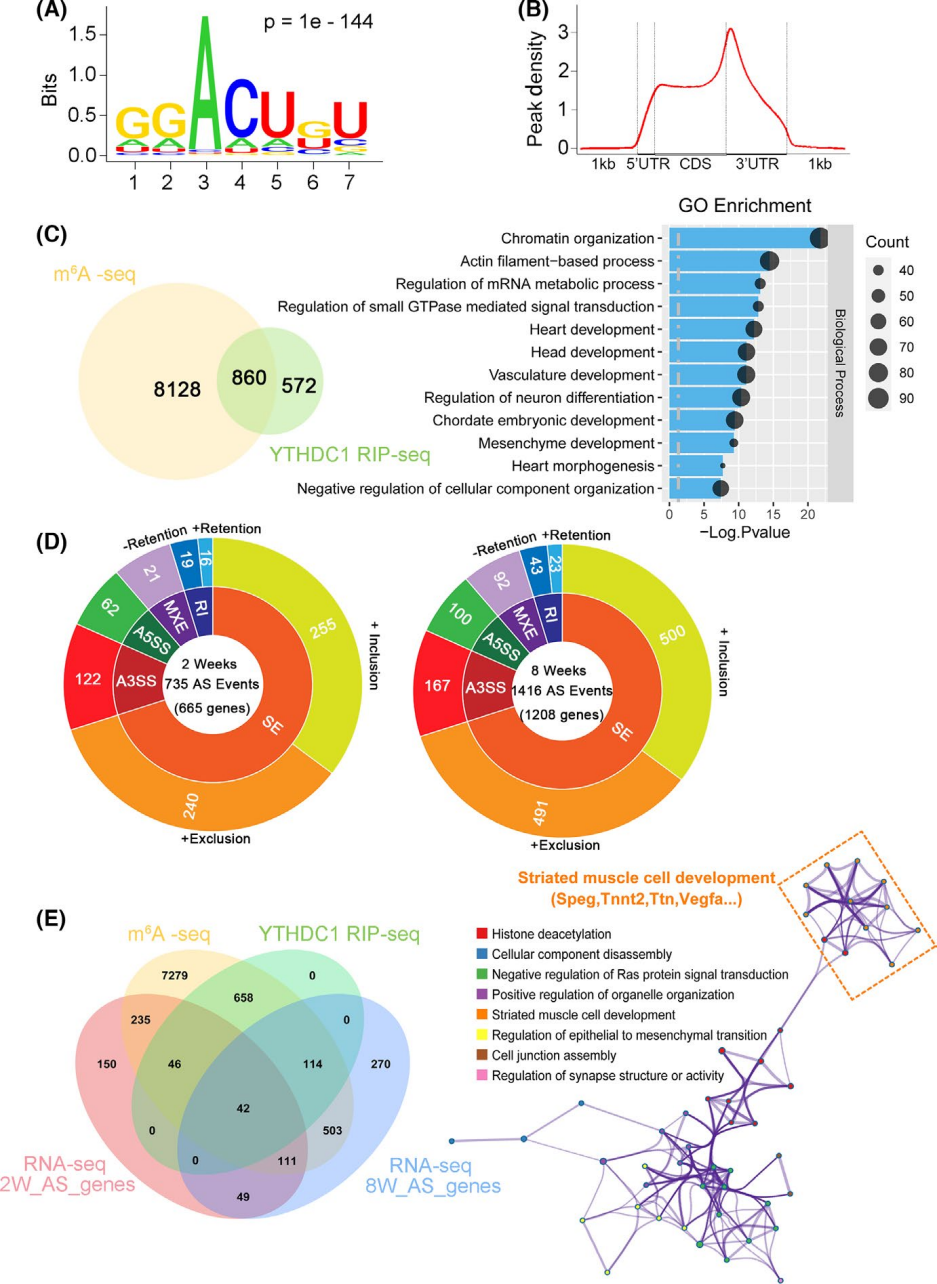
Titin is identified as a direct downstream target of YTHDC1. (A) Consensus motif of YTHDC1‐binding sites identified by HOMER of m^6^A‐seq of *Ythdc1* in mouse hearts (*n* = 2). (B) Distribution of m^6^A peaks across mRNA transcripts. (C) Left, overlap of YTHDC1 RIP‐seq genes and genes containing m^6^A in mouse hearts. Right, the top 12 GO categories enriched for YTHDC1 mRNA targets with m^6^A modification. (D) Differential alternative splicing events between Ctrl and *Ythdc1*‐*cKO* hearts at both 2‐week‐old age (left, *n* = 3) and 8‐week‐old age (right, *n* = 2) with the indicated cut‐offs. There are 735 and 1416 AS events in the 2‐week‐old age and 8‐week‐old age mice ventricle, respectively, and exon skipping is the most common alternative splicing event in both 2‐week and 8‐week mice. SE, skipped or cassette exon; A5SS, alternative 5 splice sites; A3SS, alternative 3 splice sites; MXE, mutually exclusive exons; RI, retention of introns. (E) Left, Venn diagram showing the overlap amongst m^6^A‐seq peaks (yellow), YTHDC1 RIP‐seq targets (green) and *Ythdc1*‐induced alternative splicing events from mouse hearts (red, two weeks of age; blue, eight weeks of age). Right, GO analysis showed enrichment for these genes encoding proteins involved in striated muscle cell development

We next sought to investigate the molecular mechanism by which *Ythdc1* deficiency induces the pathological phenotype of DCM. We first carried out m^6^A‐seq of wild‐type mice to obtain high‐confidence m^6^A profile of mRNA in heart. Consistent with previous studies,^4^ the conserved DRACH (R = G/A, H= U/C/A) sequence was the most enriched motif in m^6^A immunoprecipitated RNAs (*p* = 1e‐144) (Figure 4A), and m^6^A peaks were significantly distributed at start codon, stop codon and 3′ UTR (Figure 4B, Figure S5A). Then, we performed YTHDC1 RIP‐seq with mice at 2 weeks of age to identify YTHDC1‐binding targets in heart and found about 860 hits overlapped with transcripts identified by m^6^A‐seq which were defined as direct downstream targets of YTHDC1 (Figure 4C). GO analysis showed that these genes were mainly enriched in chromatin organisation, actin filament‐based process, heart development and heart morphogenesis (Figure 4C).

Given that AS is an important function of YTHDC1,^16^ we then performed mRNA‐seq to identify the AS difference of the heart tissues between Ctrl and cKO mice. To avoid the altered transcriptional regulation caused by end‐stage heart failure, we carried out the sequencing with mice at 2 and 8 weeks of age and analysed the overlapping dysregulated genes. By using rMATS, aberrant splicing targets of *Ythdc1*‐cKO were obtained for further functional characterizations (Figure 4D).

Taking these analyses together, we narrowed down targets to 42 high‐confidence transcripts, which might be potential key genes in the development of YTHDC1‐dependent DCM (Figure 4E). GO analysis revealed enrichment of these genes involved in striated muscle cell development. More importantly, we found TTN, whose truncating mutations are the most frequent cause of DCM,^42^ exists in YTHDC1‐dependent gene list. Furthermore, we detected abundant m^6^A modification in the mRNA of *Titin*, suggesting a potential role of YTHDC1 in regulation of *Titin* (Figure S5B). Then, we mutated the m^6^A binding sites (W377A, W428A) of *Ythdc1* ^17^ and found that the m^6^A binding sites mutation of *Ythdc1* completely disrupt its binding to *Titin* (Figure S6A–S6C). Thus, these data indicated that m^6^A‐modified *Titin* transcripts are likely to be regulated by YTHDC1 through an m^6^A‐dependent role.

### 3.5 YTHDC1 deficiency increases the expression ratio of N2BA to N2B isoform of *Titin*

TITIN is a sarcomeric protein that determines the structure and biomechanical properties of striated muscle, and its defect is directly associated with DCM.^43^ In the myocardium, its differential splicing leads to the expression of N2B and N2BA isoforms that differ in size.^26^ Smaller mammals express predominantly N2B, whereas both N2B and N2BA are readily detectable in larger mammals, including humans.^44^ Because of its shorter extensible I‐band region, dominant expression of N2B results in higher passive myocardial stiffness than that of N2BA. Therefore, from RNA‐Seq data, we further performed extensive analysis of alternative exon regulation of *Titin* with the Percent Spliced In (PSI) plot, showing the mean exon inclusion and exclusion.

**FIGURE 5:**
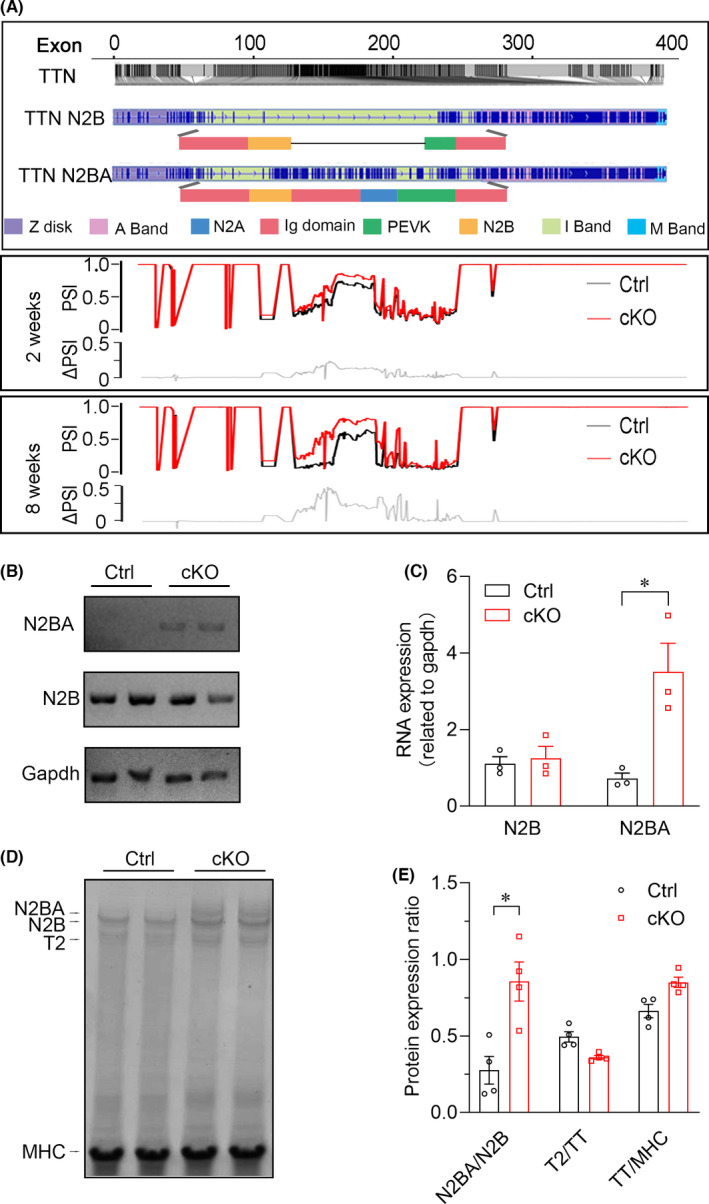
YTHDC1 deficiency increased the expression ratio of N2BA to N2B isoform. (A) Relative m^6^A intensity on TTN is shown in deep grey line. The average PSI scores for the *Ythdc1* deficient mice are indicated in red and those for control subjects are indicated in black. The ΔPSI values (grey line) show highly conserved deflections up (additional inclusion of exons) and down (exon skipping) in differentially spliced regions across ages. Splice isoform diversity in the extensible regions of N2B and N2BA titin isoforms. (B) Mean levels and error estimates for Titin N2B and N2BA isoform mRNA expression in Ctrl and *Ythdc1*‐cKO group. *Gapdh* were amplified as controls. (C) Pooled data from B demonstrating that only the Titin N2BA isoform‐specific exon was highly expressed in the homozygous mutant. N2B isoform observed no significant difference between Ctrl and *Ythdc1*‐cKO group. The data are shown as means ±SEM. *n* = 3. * *p* < 0.01 compared with ctrl group by unpaired *t* test. (D) SDS‐agarose gel electrophoresis of protein lysates from control and *Ythdc1*‐cKO left ventricular myocardium, then gel was stained with Coomassie blue. N2BA bands are broader in *Ythdc1*‐cKO samples than in controls. T2 is a minor degradation product. (E) Pooled data from D showing the ratio of N2BA to N2B, T2 to TT and TT to MHC from left ventricular homogenates (*p* < 0.001). Data are means ±SEM. *p < 0.01 compared with ctrl by unpaired *t* test

Our data illustrated that *Titin* in cKO mice was predominantly spliced in the elastic region extending from the N2B to the PEVK segments, between which the N2A signalling domain is located (Figure 5A). The alternatively spliced exons reside in the elastic PEVK and the immunoglobulin‐rich region within the I‐band, which may help to explain the increased distensibility of sarcomeres in YTHDC1 deficiency cardiomyocytes.^26^

These findings implicated that loss of *Ythdc1* induces aberrant splicing of *Titin*, leading to an increased ratio of N2BA:N2B isoform, which can directly result in DCM. We then examined the expression of the N2BA and N2B RNA isoforms in heart tissues from YTHDC1 deficiency mice and control by qRT‐PCR. As illustrated in Figure 5B, 5C in agreement with the deep sequencing results, the N2BA isoform was highly expressed in YTHDC1 deficiency hearts, whereas no difference was observed in N2B isoform. In addition, high‐resolution SDS‐agarose gels also revealed that the ratio of N2BA:N2B protein in YTHDC1 deficiency hearts was dramatically higher than that in controls due to an increase in the more compliant N2BA isoform (Figure 5D–5E). The ratios of total *Titin* (TT) to myosin heavy chain (MHC) were not different between the Ctrl and YTHDC1 deficiency hearts indicating that there was no change in the number of TITIN molecules.

### 3.6 YTHDC1 deficiency destructed the myofilament

**FIGURE 6:**
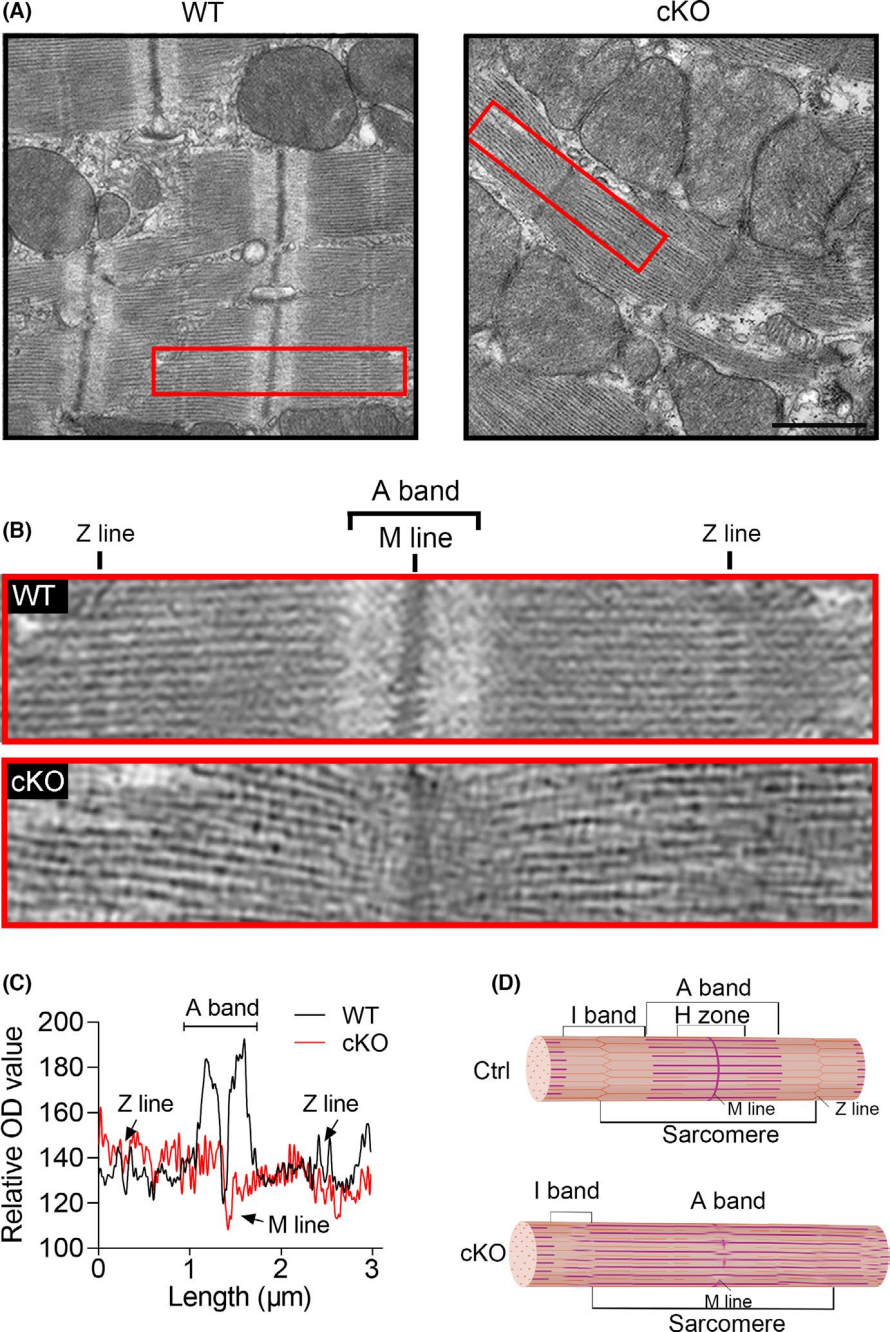
YTHDC1 deficiency destroyed the ultrastructure of the cardiac sarcomere. (A)–(B) Representative transmission electron microscopy (TEM) of sarcomere structure (top, scale bar: 1μm) from Ctrl and cKO mice at 8‐week‐old. The red box indicates an intact sarcomere. (C) The optical density (OD) analysis for the single sarcomere showing that *Ythdc1* deficiency destroyed the normal ultrastructure of the cardiac sarcomere. (D) The schematic representation of a cardiac sarcomere. The lateral boundaries of the sarcomere are the Z‐discs. The I‐bands surrounds the Z‐disc and is a region where thin filaments are not superimposed by thick filaments. The A‐band region contains thin filaments and thick filaments. The M‐band falls within the H‐zone, where thick filaments do not interdigitate with thick filaments

The sarcomere is the basic contractile unit of cardiac myofilament, which is divided into ‘I’ and ‘A’ bands, ‘M’ and ‘Z’ lines, and ‘H’ zone. And previous studies have proved that TITIN extends from the Z disc to the M‐line and the majority of its I‐band region functions as a molecular spring, contributing to the development of DCM. Thus, we further detected the ultrastructure of the cardiomyocytes by using the TEM to support the link between abnormal *Titin* splicing and DCM in *Ythdc1*‐cKO. As illustrated in Figure 6A–6B, we observed a significant disorder of filaments in the ventricular myocardium. Additionally, the optical density analysis for the TEM image showed that although no apparent defects of the primary organisation of thick and thin filaments were observed, the sarcomere units were dramatically disrupted, light I‐bands partially disappeared and the Z‐discs were blurry upon *Ythdc1* knockout in cardiomyocyte (Figure 6C–6D). These results further confirmed the impairment of cardiac contraction in YTHDC1 deficiency mice is *Titin* splicing‐mediated and suggested that YTHDC1‐depended *Titin* splicing was crucial for the maintenance of normal contractile apparatus in single cardiomyocytes and of normal heart contraction.

Taken together, the data demonstrated that *Titin* is the direct target of YTHDC1, and knockout of *Ythdc1* in heart resulted in abnormal splicing of *Titin* contributing to disarray of sarcomere structures in the cardiomyocytes, a typical feature of DCM.

## 4 DISCUSSION

We demonstrated an essential role of YTHDC1 in cardiomyocytes for the maintenance of normal heart function, with loss of YTHDC1 leading to DCM. First, the *Ythdc1*‐cKO mice exhibit early DCM, which ultimately proceeds to heart failure and postnatal lethality. Second, the *Ythdc1*‐cKO cardiomyocytes manifested disrupted myofilament and abnormal contraction function. Third, genome‐wide transcriptomic analysis revealed a subset of 42 YTHDC1‐targeted genes, amongst which *Titin* was aberrantly spliced in YTHDC1 deficiency cardiomyocytes. The discovery of *Titin* as the splicing target of YTHDC1 expanded our knowledge regarding the biological functions of the post‐transcriptional modification in DCM.

Previous studies have documented the association between the mutation of these genes and DCM; however, their post‐transcriptional regulation, especially m^6^A modification, has never been investigated.^24^ Our data suggest that the post‐transcriptional modification of a DCM‐related gene, *Titin*, may be one of the mechanisms that contribute to DCM. AS is a major feature of TTN and produces two major isoforms: N2B and N2BA. The component proportion ratio decided the lengths of the extensible I‐band domains and the contractile force of cardiomyocyte. In healthy hearts, the *Titin* N2B isoform is predominant and the *Titin* N2BA isoform is less expressed and the N2BA has less passive stiffness compared to N2B due to its larger size and increased elasticity.^45^ It has been reported that an increased ratio of N2BA:N2B isoform of *Titin* can directly result in DCM.^26^ However, the regulator that determines *Titin* splicing remains largely unknown. Recent study has identified RNA binding protein 20 as a splicing factor of TTN both in human and rat,^46^, ^47^ which again emphasises the key role of post‐transcriptional regulation in cardiac function. Our study suggested a totally novel role of YTHDC1 in *Titin* splicing regulation, which supplemented the mode of TTN regulation.

Thus far, the importance of reversible m^6^A modifications on regulation of gene expression has been unveiled in different systems.^48^ YTHDF2 modulates neural development by promoting m^6^A‐dependent degradation of neural development‐related mRNA targets.^49^ Paris et al. found that inhibition of YTHDF2 specifically compromises the propagation of leukemic stem cells.^50^ Chang et al.^51^ reported that YTHDF3 overexpression is associated with brain metastasis of breast tumour, resulting in poor survival. However, as an important member in the m^6^A readers, the role YTHDC1 in heart is not clear. Our studies reported YTHDC1 participates in the onset and progression of DCM, providing new evidence that RNA modification involves in the processing of heart disease.

DCM is one of the most common causes of heart failure.^52^ For patients with established DCM, treatment is directed at alleviating the major clinical manifestations of heart failure and arrhythmias. However, management of heart failure remains an unmet need.^23^, ^53^ Our study demonstrated that YTHDC1‐depended *Titin* splicing is crucial for the postnatal heart development and normal cardiac function, which probably provides a potential target for treating DCM through tuning m^6^A modification of *Titin* mRNA. Unfortunately, due to the limitation of single‐base resolution m^6^A sequencing technique, we did not identify the precise m^6^A site of *Titin*. Meanwhile, we did not completely exclude m^6^A‐independent pathway, such as chromatin organisation, which might also conduct the *Ythdc1* deficiency‐induced DCM. It will be of great interest to determine whether the dysfunctional *Ythdc1* gene is correlated with cardiac malfunction in human, which may facilitate the development of new therapeutic strategies for reversing DCM.

In summary, this study highlights the close relationship between the homeostasis imbalance of mRNA m^6^A modification and the occurrence and development of DCM. YTHDC1‐dependent *Titin* splicing is a potential new therapeutic target for DCM.

## CONFLICT OF INTEREST

The authors confirm that there are no conflicts of interest.

## AUTHOR CONTRIBUTIONS

**Siyun Gao:** Data curation (equal); Methodology (equal); Software (equal); Writing‐original draft (equal). **Haifeng Sun:** Formal analysis (equal); Methodology (equal); Software (equal). **Kejing Chen:** Methodology (equal). **Xueying Gu:** Methodology (equal); Software (equal). **Hongyu Chen:** Data curation (equal); Methodology (equal). **Liudan Jiang:** Methodology (equal); Software (equal). **Lei Chen:** Methodology (equal). **Shengqi Zhang:** Methodology (equal); Software (equal). **Yi Liu:** Investigation (equal). **Dan Shi:** Investigation (equal). **Dandan Liang:** Investigation (equal). **Liang Xu:** Investigation (equal). **Jian Yang:** Investigation (equal). **Yanjiao Ruan:** Methodology (equal); Software (equal). **Hao Chen:** Formal analysis (equal); Software (equal). **Bin Shen:** Conceptualization (equal); Funding acquisition (equal); Investigation (equal). **Honghui Ma:** Conceptualization (equal); Funding acquisition (equal); Investigation (equal); Supervision (equal). **Yi‐Han Chen:** Conceptualization (equal); Funding acquisition (equal); Investigation (equal).

## Supporting information

Fig S1
Fig S2
Fig S3
Fig S4
Fig S5
Fig S6

Table S1
