# Supplementary figures and images for: Depletion of m6A reader protein YTHDC1 induces dilated cardiomyopathy by abnormal splicing of Titin
Source: J Cell Mol Med. 2021 Oct 30;25(23):10879–91. doi: 10.1111/jcmm.16955 (PMC8642692; doi:10.1111/jcmm.16955)

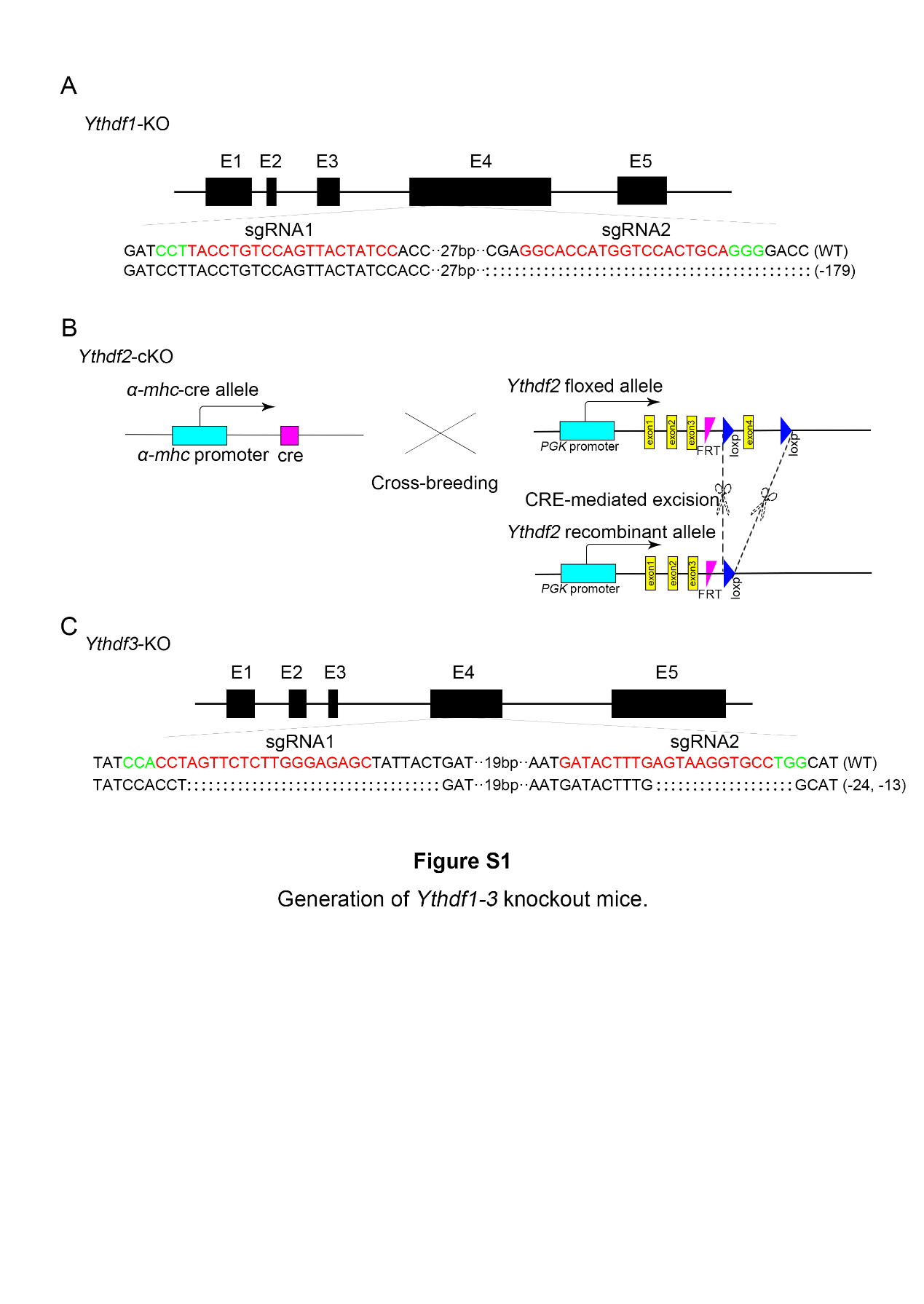

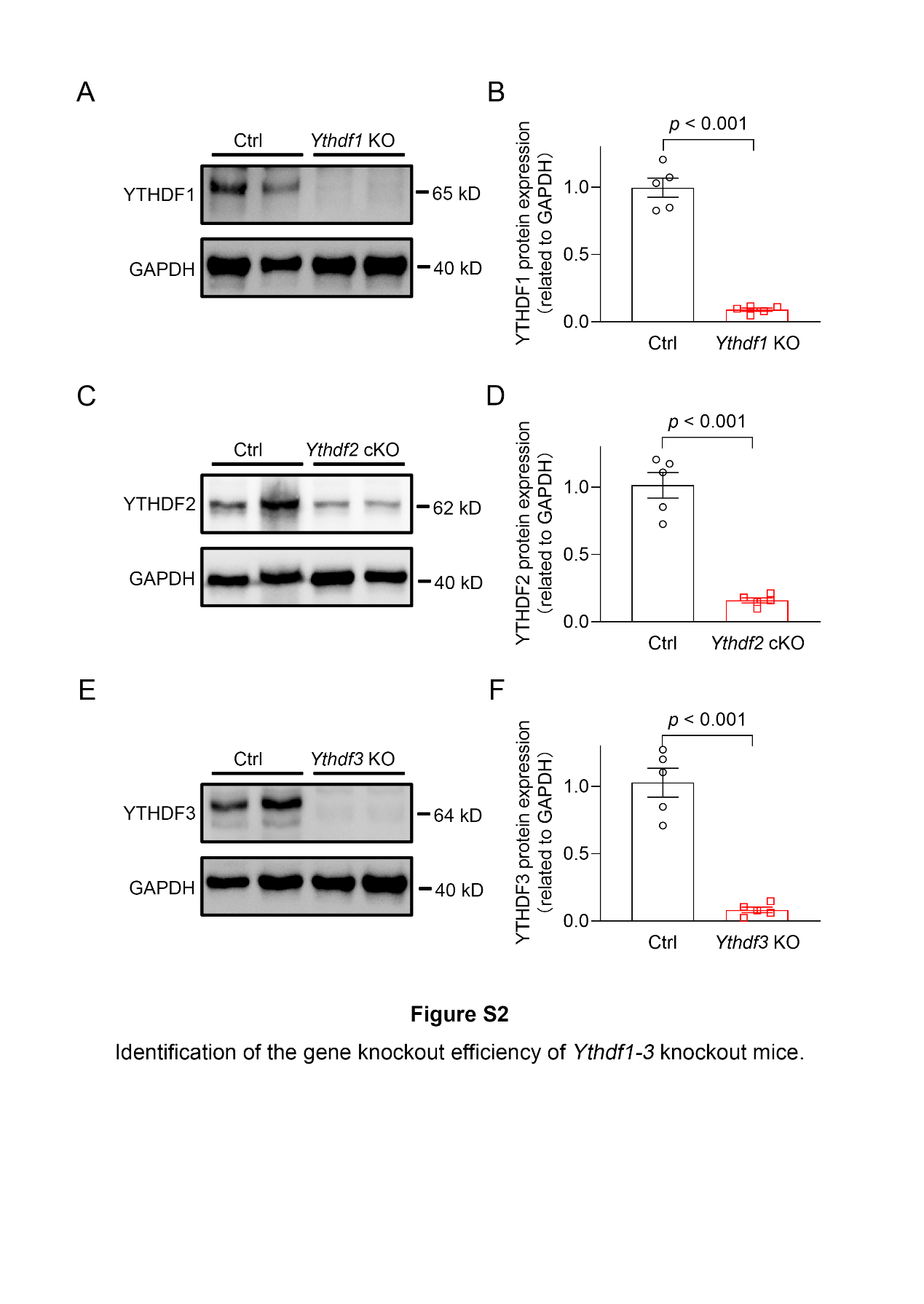

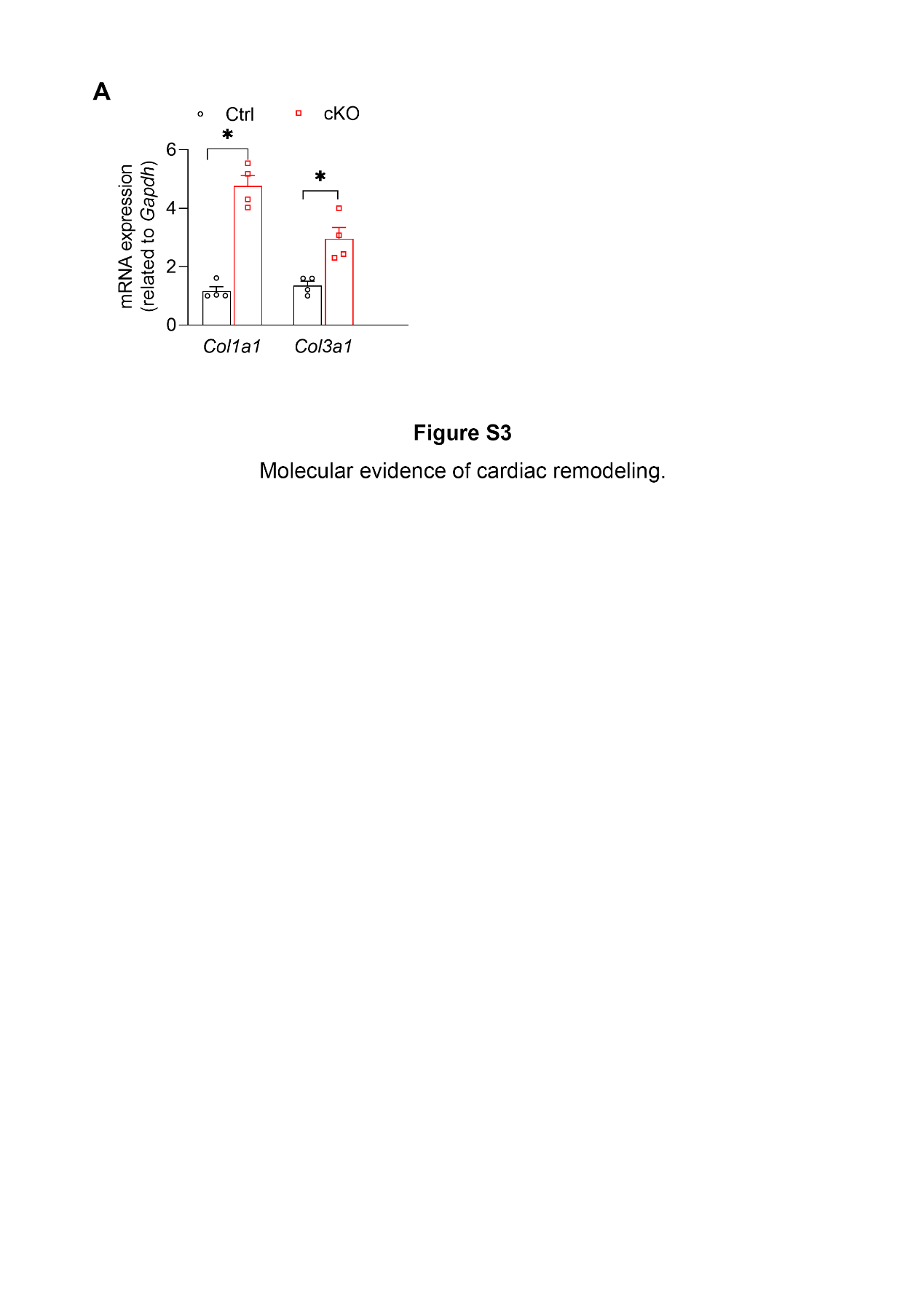

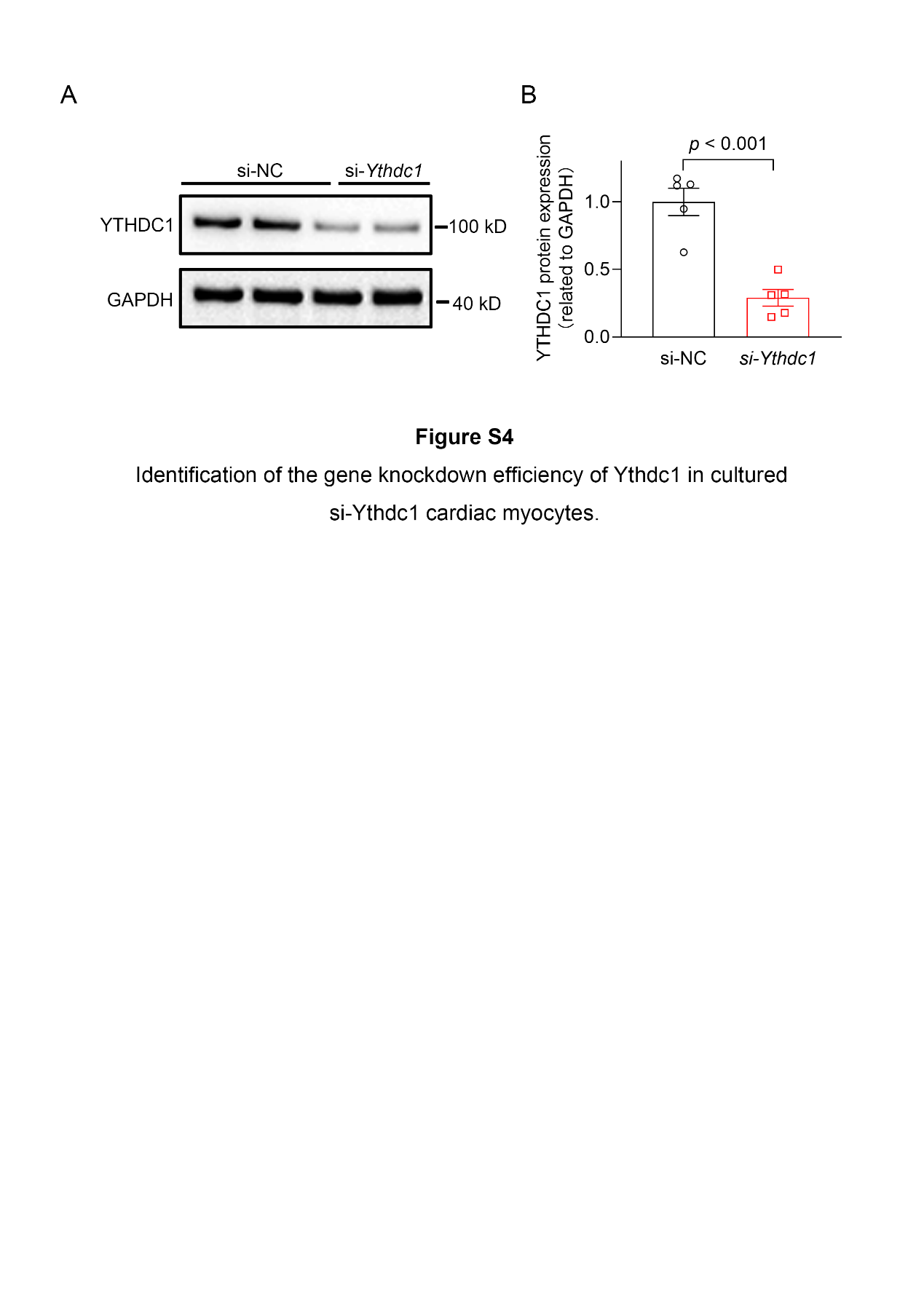

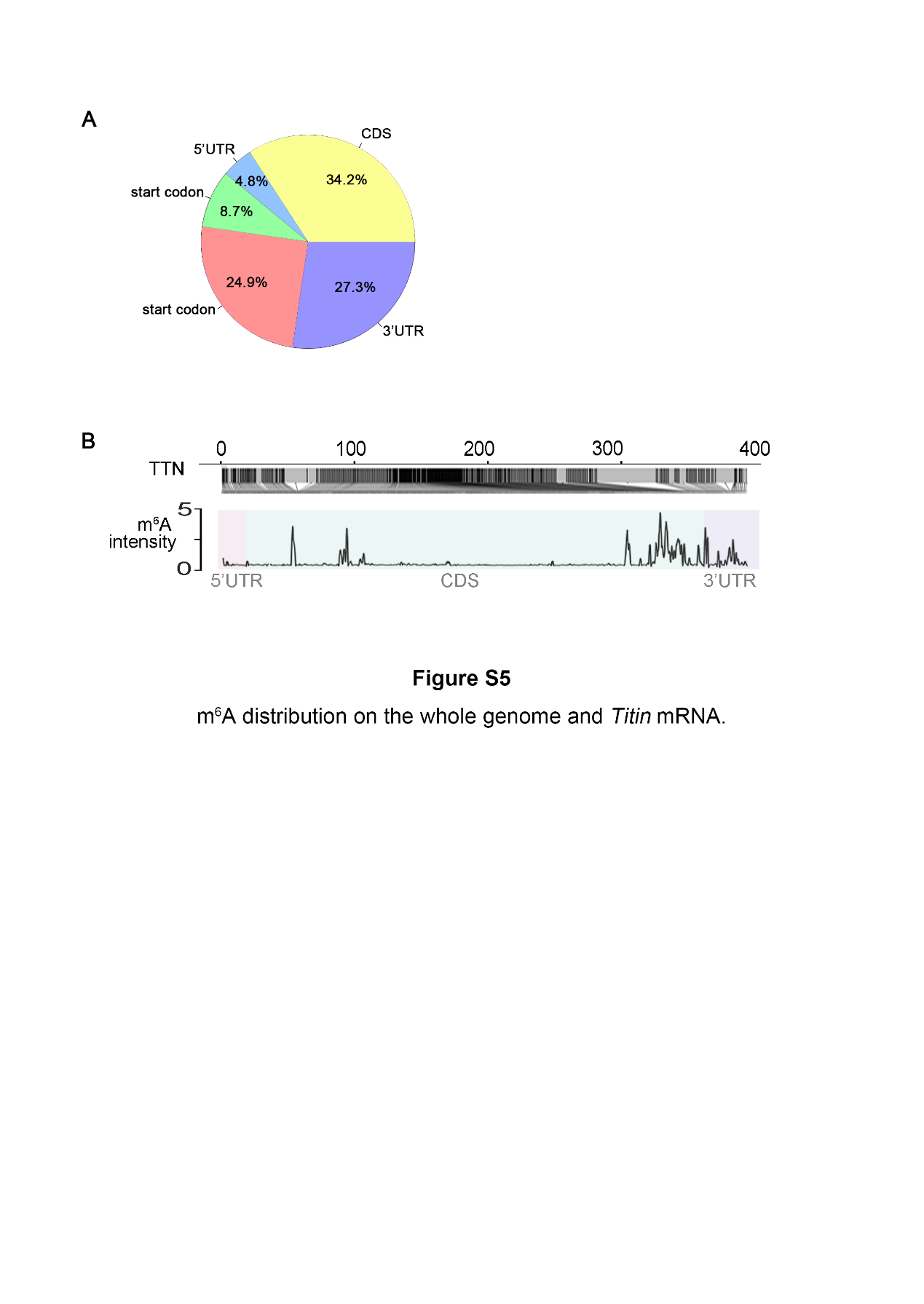

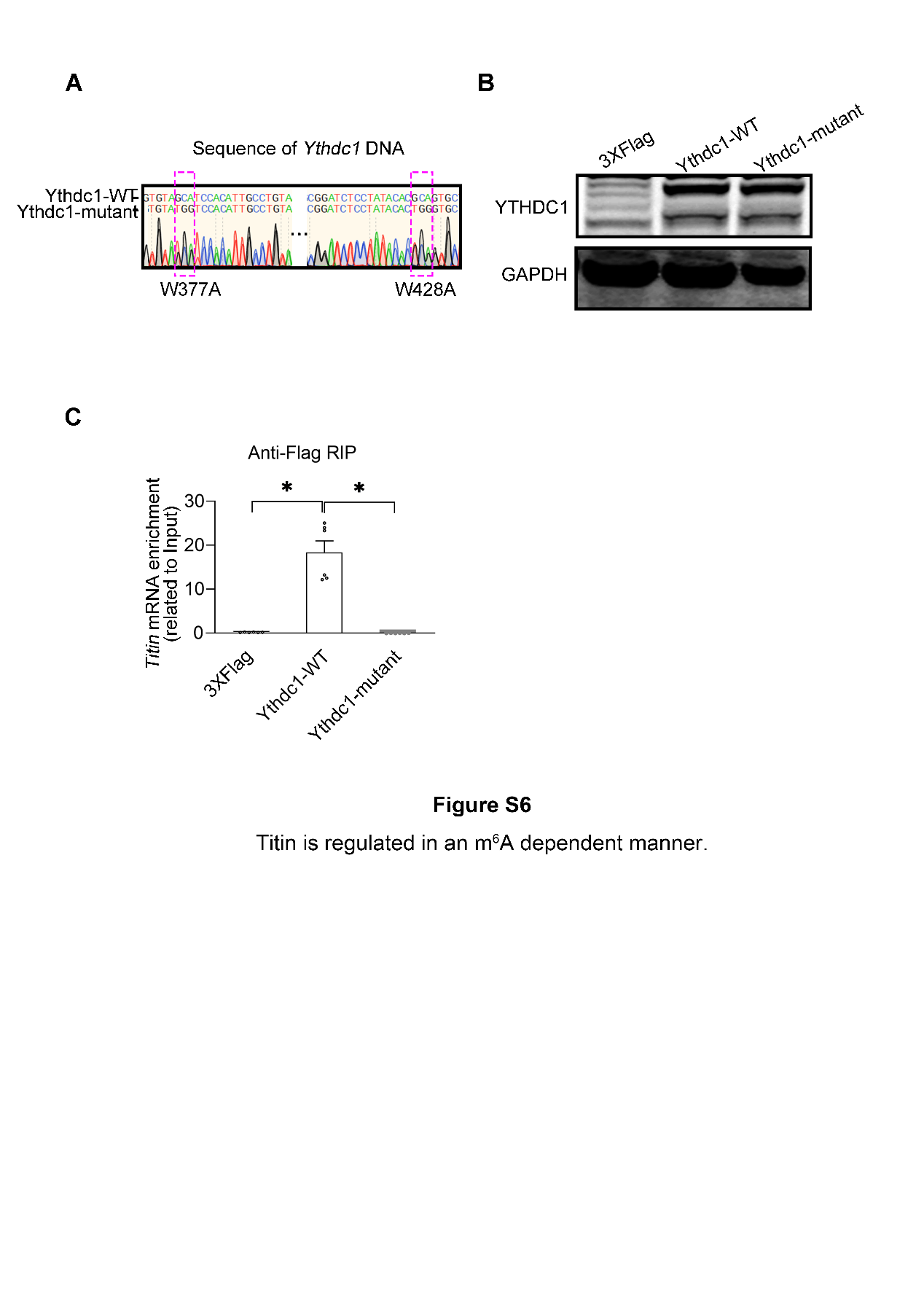

Supplement: Supplementary file 1 — Fig S1 Fig S2 Fig S3 Fig S4 Fig S5 Fig S6 [file JCMM-25-10879-s001.docx]
