# Supplementary material for: Depletion of m6A reader protein YTHDC1 induces dilated cardiomyopathy by abnormal splicing of Titin
Source: J Cell Mol Med. 2021 Oct 30;25(23):10879–91. doi: 10.1111/jcmm.16955 (PMC8642692; doi:10.1111/jcmm.16955)
Supplement: Supplementary file 2 — Table S1 [file JCMM-25-10879-s002.docx]

**Table S1: Primer sequences for PCR**

| **Name** | **Forward Primer** | **Reverse Primer** |
| --- | --- | --- |
| α-MHC-CRE | AATGCTTCTGTCCGTTTGC | ACCAGAGTCATCCTTAGCG |
| Ythdc1 | CATCTCTCCAGCCCGGTAAA | GTGCTACACTAAGTCCTGTGAC |
| N2B | ACTTCCTGAACCTAAGCCGC | TGGCTTTGGTTCAGGTCCAG |
| N2BA | AGGAGAATTGAGCCCCTGGA | GGCCTGGAGAGAAAGGTTGG |
| Gapdh | AGGTCGGTGTGAACGGATTTG | TGTAGACCATGTAGTTGAGGTCA |
| Col1a1 | CTTCACCTACAGCACCCTTGTG | CTTGGTGGTTTTGTATTCGATGAC |
| Col3a1 | TCAAGGCTGAAGGAAACAGCA | GATGGGTAGTCTCATTGCC |
